# Gut dysbacteriosis attenuates resistance to *Mycobacterium bovis* infection by decreasing cyclooxygenase 2 to inhibit endoplasmic reticulum stress

**DOI:** 10.1080/22221751.2022.2096486

**Published:** 2022-07-21

**Authors:** Haoran Wang, Jiao Yao, Yulan Chen, Yuanzhi Wang, Yiduo Liu, Yi Liao, Zhengmin Liang, Yu hui Dong, Mengjin Qu, Xin Ge, Xiangmei Zhou

**Affiliations:** a College of Veterinary Medicine, China Agricultural University, Beijing, People’s Republic of China; b College of Animal and Veterinary Sciences, Southwest Minzu University, Chengdu, People’s Republic of China.

**Keywords:** Gut dysbacteriosis, cyclooxygenase 2, *Mycobacterium bovis*, endoplasmic reticulum stress, apoptosis, faecal transplant

## Abstract

The role of gut microbiota has been described as an important influencer of the immune system. Gut-lung axis is critical in the prevention of mycobacterium infection, but the specific mechanism, by which dysbiosis affects tuberculosis, has not been reported. In this study, we attempted to provide more information on how the gut-lung axis contributes to *Mycobacterium bovis* (*M. bovis*) infection. Mice are pre-treated with broad-spectrum antibiotics cocktail (Abx) to induce gut dysbiosis. Interestingly, dysbiosis of microbes showed a significant increase in the bacterial burden in the lungs and inhibited the level of COX-2. After faecal transplantation, cyclooxygenase 2 (COX-2) expression was restored and the inflammatory lesion in the lungs was reduced. Further research found that the deficiency of COX-2 inhibited endoplasmic reticulum stress (ER stress). This mechanism was completed by COX-2 interaction with BIP. Moreover, we found a positive feedback mechanism by which blocking ER stress could reduce COX-2 levels by the NF-κB pathway. Taken together, we reveal for the first time gut dysbacteriosis exacerbates *M. bovis* disease by limiting the COX-2/ER stress pathway. The finding strengthens the foundation of gut microbiota-targeted therapy for tuberculosis treatment.

## Introduction

Bovine tuberculosis (bTB) is a zoonotic disease caused by *Mycobacterium bovis* (*M. bovis*). As an OIE-listed disease, bTB must be reported to the OIE following the Terrestrial Animal Health Code [1]. It not only causes animal tuberculosis but also infects humans through inhaling contaminated aerosol and ingesting unpasteurized milk products from animal carriers. [2]. Host gut microbiota or metabolites derived from them can be vital factors in lungs’ immune response to *Mycobacterium tuberculosis* (*Mtb)* infection [3,4]. Gut dysbacteriosis affects gut inflammation by influencing the release of pro-inflammatory/anti-inflammatory cytokines or metabolites and several extraintestinal diseases, such as atopic dermatitis, allergy, obesity, and diabetes [5–7]. In recent years, increasing studies reported that gut dysbacteriosis promotes macrophage M2 polarization and allergic airway inflammation [8]. Some bacterial metabolites such as short-chain fatty acids (SCFAs) are known to be immunoreactive that upregulate the immune system in response to pulmonary pathogens [9–11]. However, it is still unknown the specific mechanism, by which the gut microbiome affects *Mtb* infection.

Endoplasmic reticulum (ER) stress results from the accumulation of unfolded and misfolded proteins within ER lumen induced by hypoxia or nutrient deprivation (UPR) [12]. When UPR occurs, glucose-regulated protein 78 (BIP) dissociates from ER transmembrane proteins, including PKR-like ER kinase (PERK), inositol-requiring enzyme 1 (IRE1), and activating transcription factor 6 (ATF6) induces ER stress to prevent unfolded protein accumulation and maintain intracellular homeostasis [13–15]. Previous studies show that ER stress plays an important role in the body's defence against intracellular bacterium infection by activating apoptosis [16,17]. However, whether ER stress is involved in the interaction between gut dysbacteriosis and *M. bovis* infection remains to be determined.

*Mtb* infection results in increased expression of cyclooxygenase-2 (COX-2), a rate-limiting enzyme in the arachidonic acid (AA) pathway [18,19]. It is an inducible enzyme that is induced by different stimuli and has a highly restricted expression pattern [20]. COX-2 has enzymatic actions on AA, which could oxidize AA to form prostaglandin E2 (PGE2), a pro-inflammatory factor in the host immune system [21]. For instance, during *L. monocytogenes* infection, macrophages and dendritic cells resist infection by producing COX-2, which activates CD8^+^ T cells [22].

Currently, COX-2 is discovered to aid the antimycobacterial immune response through four different mechanisms. First, without the protective actions of COX-2, the *Mtb*-infected macrophage is more likely to undergo necrosis by activating lipoxin A4 (LXA4). Necrosis allows the *Mtb* to evade innate immunity [23,24]. Second, membrane micro-disruption occurs during infection with attenuated *Mtb*. COX-2 expression promotes the repair of disrupted plasma membranes and prevents host cell necrosis [25]. Third, COX-2 inhibits mycobacteria proliferation by increasing autophagy by the AKT/mTOR pathway [26]. Finally, COX-2 catalysis promoted by interleukin 1(IL-1β) yields increased levels of the reaction product PGE2, which cooperates with IL-1β to limit excessive type I interferon (IFN) production and prevent the death of *Mtb*-infected mice during the acute phase of infection [27]. Although COX-2 and ER stress play a critical role in macrophage-mediated immune responses to mycobacteria, there is no evidence of the association of COX-2 with ER stress during mycobacterial infection.

Intestinal flora disorder can affect innate immunity and play a key role in the control of TB infection. In addition, our previous study suggests that ER stress plays an important role in defencing *M. bovis* infection [17]. Therefore, we raised the following questions: (1) Do gut dysbacteriosis affect ER stress with *M. bovis* infection? (2) If so, how does it affect and (3) what is the mechanism?

In this study, we found that gut dysbacteriosis aggravated *M. bovis* infection and reduced the levels of COX-2 and PGE2 yield. Next, we observed the inhibition of COX-2 by celecoxib increased the lung bacterial burden of *M. bovis*-infected mice and reduced ER stress. Furthermore, we demonstrated that COX-2 regulated ER stress, which was caused by the association of COX-2 with BIP. Meanwhile, we discovered a positive feedback mechanism by which blocking ER stress could reduce COX-2 levels by the NF-κB pathway. Collectively, these results indicate that the inhibition of the COX-2/ER stress pathway might be one of the strategies for gut dysbacteriosis affecting *M. bovis* infection. The findings indicate that gut microbiota-targeted therapy could be a novel approach to *M. bovis* infection control.

## Materials and methods

### Animals and ethical statement

A total of 60 six- to eight-week-old female C57BL/6 mice were purchased from SPF Biotechnology (Beijing, China) and housed in cages under the BSL3 laboratory facilities of China Agricultural University. Mice were housed in standard cages under conventional conditions (at a temperature of 21 ± 1°C, relative humidity of 50 ± 10%) with a regular 12:12-h light: dark cycle with the light on at 8:00 AM. All the mice had free access to food and water, with the same amount in each cage.

Animal experiments were conducted following protocols approved by the animal care and use committee (IACUC) of China Agricultural University, Beijing, and following the regulations for the care of laboratory animals established by the Ministry of Science and Technology People’s Republic of China. The procedures of the present animal study were reviewed and approved by The Laboratory Animal Ethical Committee of China Agricultural University, Beijing, China, under approval number 20110611–01. Experiments on *M. bovis* culture and animal infection were conducted in strict biosafety conditions in BSL-3 laboratory facilities in the NTSE laboratory of China Agricultural University, Beijing.

### Animal models for dysbiosis and *M. bovis* infection

Mice were randomly assigned into six experimental groups: (1) Control group (CT); (2) Abx-treated group (Abx); (3) Abx and faecal transplants-treated group (Abx + FT); (4) *M. bovis*-infected group (*M. bovis*); (5) *M. bovis* with Abx-treated group (Abx + *M. bovis*); (6) *M. bovis* with Abx and faecal transplants-treated group (Abx + FT + *M. bovis*). Mice were given a combination of broad-spectrum antibiotics ad libitum (Abx) in drinking water for three weeks, which included ampicillin (1 g/L, Solarbio, Beijing, China), vancomycin (500 mg/L, Solarbio, Beijing, China), and neomycin sulphate (1 g/L, Solarbio, Beijing, China), and this treatment was supplemented with metronidazole from the fourth (0.5 g/L, Solarbio, Beijing, China) to seventh (1 g/L) week. Abx water was changed once a week. Mice were anesthetized using Zoletil (50 mg/kg) with *M. bovis* C68004 strain, provided by the China Institute of Veterinary Drug Control (CVCC, China), via the intranasal route (i.n.) with 200 CFU after 3 weeks of Abx treatment. *M. bovis* was grown in a 7H9 liquid medium with OADC (BD, USA) (Supplement Figure 1A). The mice were continuously treated with Abx for 5 weeks after infection and then sacrificed. Next, using sterile sampling containers, fresh faecal pellets were collected immediately from the colonic tissue of each mouse (all animals were in the agonal stage). The faecal pellets were immediately frozen in liquid nitrogen and transported to the laboratory.

### Microbiota restoration

The stool was collected from untreated mice (200–300 mg) and immediately homogenized in PBS (1 mL). The solution was then centrifuged at 2000 rpm for 2 min, the supernatant was collected, and 0.2 mL was administered intragastrically to each mouse.

### CFU assay

For the CFU assay, lung tissues homogenize using small ceramic beads in a tissue homogenizer apparatus (WKT technology). Each sample received ten-fold dilutions in sterilized PBS, plated in triplicate on 7H10 agar plates culture, and incubated for two to three weeks. CFUs of *M. bovis* bacilli were counted after 3 weeks of incubation at 37°C.

### Histopathological examination

Lung specimens from the left lobe were fixed in 10% formalin, embedded in paraffin, sectioned, and stained with haematoxylin and eosin (H&E) for histological examination. A thin section of lung tissues was stained with the Ziehl–Neelsen (Z&N) (Solarbio, Beijing, China) staining method to observe the dissemination of *M. bovis* bacilli in the lung tissues of experimental mice. The H&E and Z&N-stained sections were examined under low (10× and 20×) and high (40× and 100×) magnification and clear images were captured with an Olympus microscope camera.

### Animal models for inhibition of COX-2 with *M. bovis* infection

Mice were randomly assigned to four experimental groups: (1) Control group (*n* = 7) (CT); (2) Celecoxib (Selleck, USA) +*M. bovis* group (*n* = 7) (Cele + *M. bovis*); (3) *M. bovis* infection group (*n* = 7) (*M. bovis*); (4) Celecoxib (20 μg/kg) (Selleck, USA) + Dimethyl PGE2 (50 μg/mL per mouse) +*M. bovis* group (*n* = 7) (Cele + dm-PGE2 + *M. bovis*). Celecoxib or dm-PGE2 was administered intraperitoneally for 5 weeks. The mice were sacrificed after 5 weeks of infection. The fresh stool was collected immediately from the colonic tissue of each mouse using sterile sampling containers (Supplement Figure 1B).

### Isolation of lung cells

Excised lungs were washed with PBS and incubated on ice for 5 min. The sectioned lungs tissues were transferred to a six-well plate containing 5 mL RPMI-1640 complete medium and 50 μL collagenase 1A (1 mg/mL) and 50 μL DNase1 (150 U/mL). The tissues were digested for 1 h. The digestants were centrifuged at 1000 rpm for 5 min and the supernatant was discarded. 3 mL red blood cell lysis buffer was added and incubated for 5 min. Then, 3%FBS was added to block the reaction. Cell suspensions were centrifuged at 1000 rpm for 5 min and the sediment was resuspended by 1 mL RPMI-1640.

### Flow cytometric analysis of macrophages

Briefly, 5 × 10^6^ macrophages were put into a 1.5 mL EP tube and mixed with 1× binding buffer (500 μL). Cells were then treated with 5 μL Annexin-V FITC and 10 μL PI (Multisciences, Hangzhou, China) and incubated for 5 min. The cells were tested using BD FACS ARIA (NJ, USA) and analyzed using Flow jo software.

### ELISA assay

The expression level of PGE2 was measured using ELISA kits purchased from Mlbio (Shanghai, China). Cytokine levels were determined based on the absorbance at 450 nm measured using a microplate reader. 50 μL serum and 50 μL Biotin-antibody per well were added and incubated for 1 h at 37°C. The liquid of each well was removed and washed with PBS three times. 80 μL of HRP-avidin (1×) was added to each well and incubated for 1 h at 37°C and washed five times. 50 μL solution A and B was added and incubated for 15–30 min at 37°C. 50 μL of Stop Solution was added to each well, the plate was gently tapped to ensure thorough mixing. Cytokine levels were determined based on the absorbance at 450 nm measured using a microplate reader.

### Quantitative real-time PCR (qRT-PCR)

The total RNA was extracted from cells using TRIzol Reagent (Invitrogen, Carlsbad, CA, USA). The RNA samples were reverse-transcribed into cDNA with the RevertAid First Strand cDNA Synthesis Kit (Thermo Fisher Scientific, Waltham, MA, USA) following the manufacturer’s guidelines. The stool DNA was extracted using a stool DNA kit (OMEGA, NY, USA) following the manufacturer’s instructions. Quantitative real-time PCR was performed using the applied biosystems (Themo Fisher, NY, USA) and SYBR green mix (Vazyme, Nanjing, China). The quantitative RT–PCR data were analyzed using the comparative CT method (2^−ΔΔCT^). The GAPDH served as the internal control. The primers’ sequences used for PCR are listed in the supplement Table 1. All samples were analyzed in triplicate.

### Cell culture and infection models

RAW264.7 macrophage cells were obtained from the Cell Culture Center, Peking Union Medical College (Beijing, China) and cultured in a humidified incubator at 37°C with 5% CO_2_ in DMEM (Hyclone, Logan, UT, USA) supplemented with 10% FBS (Gibco, Grand Island, NY, USA), 100 μL/mL streptomycin, and 100 μL/mL penicillin (Gibco, Grand Island, NY, USA). Cells were transferred to 12- or 24-well culture plates in DMEM with 2% FBS and cultured for 12–18 h. For the cell infection model, *M. bovis* was added to RAW264.7 at an MOI = 10 and incubated for 3 h. Following that, the inoculum was removed, and cells were washed with PBS and cultured in a fresh medium in a CO_2_ incubator at 37°C. The first 3 h of the incubation was regarded as the phagocytosis period for macrophages and counted as 0 h post-infection. Samples were collected 24 h post-infection.

For inhibitor pre-treated samples, RAW264.7 was treated with 80 μM PDTC (Beyotime, Shanghai, China), 5 mM 4-PBA (Cayman Chemical, Ann Arbor, MI, USA.), 30 μM Celecoxib (Selleck, USA), 20 μM Zileuton (MCE, Beijing, China) separately, for 3 h before infection.

### Western blotting analysis

Lung tissues were homogenized and lysed in Radio immunoprecipitation assay (RIPA) buffer (20 mg/150 μL) (Solarbio, Beijing, China) supplemented with Phenylmethylsulfonyl fluoride (PMSF)p in a 1.5 mL EP tube. The cells were also lysed in RIPA buffer-containing PMSF (1*10^6^ cell/150 μL). The homogenized samples were centrifuged to collect the supernatants, which were mixed with a loading buffer and then boiled for 10 min. The proteins were then separated on 12% SDS-PAGE and transferred to the PVDF membrane (Millipore, Billerica, MA, USA). The membrane was incubated in COX-2 antibody (Proteintech, Wuhan, China, Catalog number 66351-1-Ig), Caspase-3 antibody (Proteintech, Wuhan, China, Catalog number: 19677-1-AP), BIP antibody (Proteintech, Wuhan, China, Catalog number: 11587-1-AP), GAPDH antibody (Proteintech, Wuhan, China, Catalog number: 10494-1-AP), α-Tublin antibody (Proteintech, Wuhan, China, Catalog number: 11224-1-AP), Histone 3 antibody (Proteintech, Wuhan, China, Catalog number: 17168-1-AP), Phospho-eIF2α antibody (Cell Signaling Technology, Boston, USA, Catalog number #3398), eIF2α antibody (Cell Signaling Technology, Boston, USA, Catalog number #5324), CHOP antibody (Cell Signaling Technology, Boston, USA, Catalog number #2895), IgG (Beyotime, Shanghai, China, Catalog number #AF1804) Phospho-p65 antibody (Cell Signaling Technology, Boston, USA, Catalog number #3031), and p65 antibody (Cell Signaling Technology, Boston, USA, Catalog number # 8242) overnight at 4℃. Subsequently, it was incubated with horseradish peroxidase-conjugated IgG secondary antibody (dilution 1: 3000) for 1 h at room temperature. The protein bands were developed and imaged using the ChemiDoc XRS system (Bio-Rad, USA). The relative density of the blots was quantified using Image J.

### CO-IP

The cells were also lysed in RIPA buffer-containing PMSF(1*10^6^ cell/150 μL). The homogenized samples were centrifuged to collect the supernatants. The cell lysate was cleared by adding 20 μL of Protein A/G-Agarose (Abmart, China, Shanghai) slurry (50%) per 1 mL of cell lysate and incubating at 4°C for 10 min on a rotator. The Protein A/G-Agarose was removed by centrifugation at 14,000 g at 4°C for 5 min. The supernatant was transferred to a fresh centrifuge tube. The 5 μL BIP or COX-2 antibody of cell lysate was added. The cell lysate/antibody mixture was gently rotated for either 2 h or overnight at 4°C on a rotator. The immunocomplex was captured by adding 30–40 μL Protein A/G-Agarose slurry (15–20 μL packed beads) and gently rotated on a rotator for 1 or 2 h at 4°C. The agarose beads were collected by centrifuging for 3 min at 1000 rpm. The supernatant was discarded and the beads were washed three times with an 800 μL ice-cold RIPA buffer. The agarose beads were resuspended in 30–60 μL 1× SDS loading buffer and mixed gently. The agarose beads were boiled for 10 min at 100°C to dissociate the immunocomplexes from the beads. The beads were collected by centrifugation and SDS-PAGE was performed with the supernatant. Unused samples may be stored at −20°C for later use.

### Confocal microscopy analysis

RAW264.7 cells were grown in 24-well plates with or without *M. bovis* for 24 h and then fixed in 4% paraformaldehyde (pH 7.2–7.4) for 20 min. The samples were incubated in PBS and 0.3% Triton X-100 for 5 min on ice. They were blocked with a blocking buffer and then subjected to immunostaining (Beyotime, Shanghai, China) for 30 min. The cells were incubated overnight at 4°C with COX-2 and BIP antibody (dilution 1: 200). After washing, the samples were incubated with goat anti-mouse/rabbit IgG secondary antibody (Alexa Fluor 488/594, Yeasen, Shanghai, China) for 1 h in the dark and then counterstained with DAPI (Beyotime, Shanghai, China) for 10 min. It was then washed using PBS three times and imaged with a confocal microscope (Olympus, Japan)

### Extraction of nuclear and cytoplasmic protein

Nuclear and cytoplasmic proteins were extracted using nuclear and cytoplasmic protein extraction kits (Beyotime, Shanghai, China) according to the manufacturer’s instructions.

### Statistical analysis

Statistical analysis was performed using Graphpad Prism 8. Data between two groups were compared with the Study’s *t*-test. Statistical significance was expressed as *p < 0.05, **p < 0.01, ***p < 0.001. A *p* < 0.05 was considered statistically significant.

## Results

### Abx treatment significantly alters the gut microbial composition and aggravates *M. bovis* infection

Gut dysbacteriosis is involved in a variety of extraintestinal diseases [5,7].

**Figure 1.**
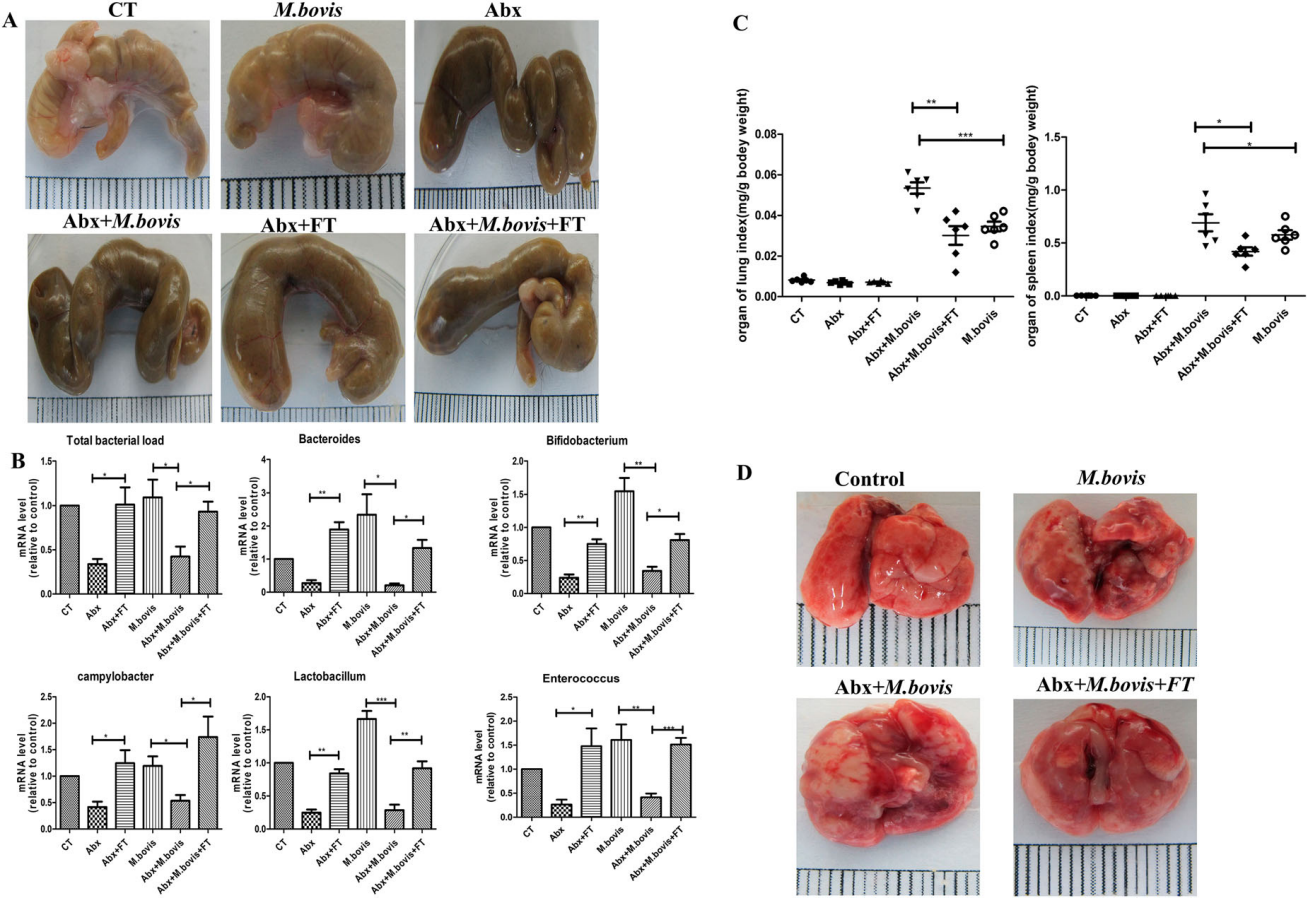
The effects of gut dysbacteriosis on the severity of *M. bovis* pathogenesis in mice. (A) The representative images of caecum showed the gross pathological changes in all experimental groups (*n* = 6). (B) Mice were pre-treated with a broad spectrum of antibiotics followed by *M. bovis* infection. Later, DNA was isolated from faecal samples of mice for quantitative PCR analysis and normalized to a universal bacterial primer. Bar graphs depict the total bacterial load; bacterial genera such as *Lactobacillus*, *Bifidobacterium*, *Campylobacter*, *Bacteroides* and *Enterococcus*. (C) The organ index of the spleen and lungs in all experimental groups. (D) The representative images of the lungs showed the gross pathological changes of all experimental groups (*n* = 6). The dots with different shapes show the number of mice and the horizontal lines indicate the mean value. Data are shown as mean ± SD, **P < 0.01. *P < 0.05.

**Figure 2.**
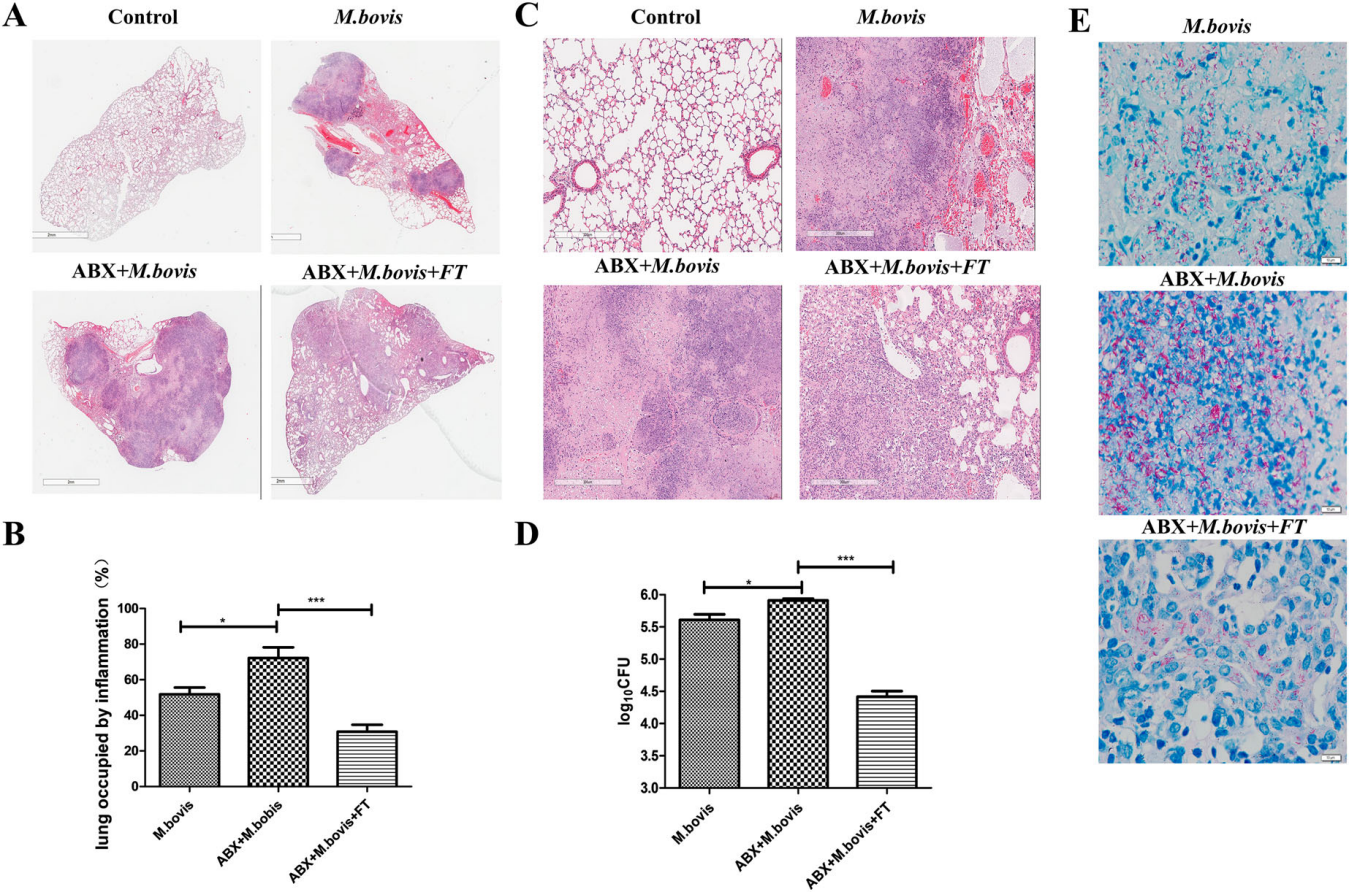
The effects of gut dysbacteriosis on histopathological changes in the lung tissues from *M. bovis*-infected mice (A) The representative images of the left lung lobe showed histopathological changes in uninfected (CT), *M. bovis*-infected mice (*M. bovis*), *M. bovis* with Abx-treated mice (Abx + *M. bovis*), *M. bovis* with Abx and faecal transplants treated group (Abx + FT + *M. bovis*). (B) The percentage of the lung’s area occupied by inflammatory lesions was quantified by Image J software. (C) Higher magnification of H&E staining sections of lungs and spleen showed *M. bovis I-*induced lesions. Scale bar: 20 μm. (D) The total number of *M. bovis bacilli* in the lung tissues of infected mice as determined using the CFU assay. (E) Ziehl–Neelsen staining results showing the number of *M. bovis* in the lungs of mice with gut dysbacteriosis. Data are shown as mean ± SD, ***P < 0.001, **P < 0.01. *P < 0.05

To investigate the effect of gut dysbacteriosis on the pathogenesis of *M. bovis* in vivo, we infected mice with *M. bovis* with or without Abx treatment. Microbial recolonization over time in Abx mice was only partial, which correlated with the presence of an enlarged caecum in Abx mice, as previously observed in Germ-free mice [28]. We observed that the caecum of Abx-treated mice has significantly dilated three weeks post-infection, which was alleviated by faecal transplantation (Figure 1(A)). Meanwhile, RT–PCR results revealed that several major species of intestinal flora decreased significantly by Abx treatment and increased by faecal transplantation (Figure 1(B)). Compared with mice without Abx, Abx-treated mice had significantly reduced weight and significantly increased spleen and lung organ index post-infection, a phenotype was reversed by faecal transplantation (Figure 1(C) and Supplement Fig. 1C). Gross pathological observation showed that Abx treatment aggravated lesions in the lungs and spleens of infected mice. Lungs with more greyish-white nodular lesions and sweller spleen were observed in Abx-treated mice with *M. bovis* infection, whereas faecal transplantation could mitigate these lesions (Figure 1(D) and Supplement Fig. 1D). Consistent with gross pathological observation, histopathological observation showed that extensive inflammatory lesions with necrosis and calcification appeared in the lungs of Abx-treated mice with *M. bovis* infection and much fewer inflammatory lesions appeared in the lungs of mice treated with faecal transplantation (Figure 2(A–C)). In addition, Abx treatment exacerbated bacterial proliferation, leading to increased bacterial burden and distribution in the lungs, which was reversed by faecal transplantation (Figure 2(D,E)). Altogether, these data showed that gut dysbacteriosis can aggravate *M. bovis* infection.

### Gut dysbacteriosis decreases COX-2 expression, ER stress, and apoptosis *in vivo*

**Figure 3.**
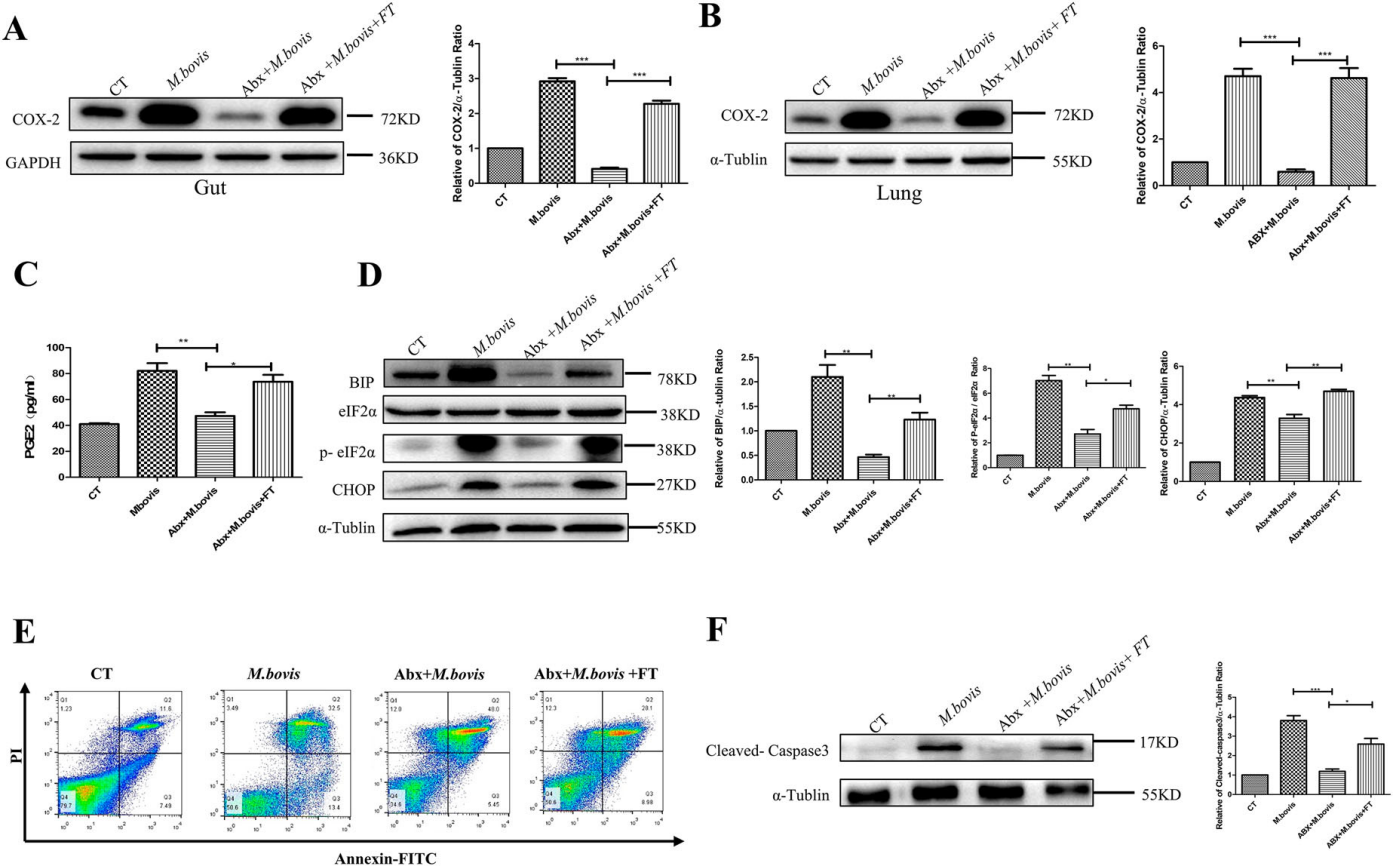
Gut dysbacteriosis inhibited the expression of COX-2, ER stress and apoptosis. (A) Western blot detection and the relative intensity ratio of COX-2 in the gut of different groups of mice. GAPDH expression served as an internal control and was used for normalization (*n* = 3/group). (B) Western blot detection and the relative lung ratio of COX-2 in the lungs of a different group of mice. α-tublin expression served as a lung control and was used for normalization (*n* = 3/group). (C) ELISA detected the alteration of PGE2 in serum. (D) Western blot detection and the relative lung ratio of BIP, p-eIF2α and CHOP in the lungs of a different group of mice. α-tublin and eIF2α expression served as a lung control and was used for normalization (*n* = 3/group). (E) macrophage isolated from the lungs of a different group of mice were treated with Annexin-V and detected by flow cytometry. (F) Western blot detection and the relative lung ratio of cleaved-caspase3 in the lungs of a different group of mice. α-tublin expression served as a lung control and was used for normalization (*n* = 3/group). Data are shown as mean ± SD, **P < 0.01. *P < 0.05.

COX-2 plays an important role in defence against *Mtb* infection [29]. Hence, we detected the COX-2 protein levels in the lung and gut using western blotting. We observed that *M. bovis* infection increased the protein level of COX-2 was inhibited by Abx treatment and increased by faecal transplantation treatment in the lungs and guts (Figure 3(A,B)). Meanwhile, the ELISA analysis revealed that Abx treatment suppressed the level of PGE2 in serum, while faecal transplantation enhanced it (Figure 3(C)). These data support the theory that gut dysbacteriosis caused a decrease in COX-2 levels throughout the body.

Disruption of ER homeostasis has been shown to activate host immunity to resist tuberculosis infection [17]. Hence, we detected the protein level of BIP, phosphorylated eIF2-α (P-eIF2α), and CHOP, which were markers of protein of ER stress. Compared with mice not treated with Abx, Abx-treated mice significantly reduced the protein level of BIP, P-eIF2α, and CHOP. Following faecal transplantation treatment, the symptom was alleviated (Figure 3(D)).

Next, we assessed the level of apoptosis by the flow cytometric analysis and western blotting. Compared with mice not treated with Abx, Abx-treated mice decreased the number of apoptotic cells and the protein level of Cleaved-caspase-3, while faecal transplantation increased them (Figure 3(E,F)). These results demonstrate that dysregulation of intestinal flora could inhibit the expression of COX-2, ER stress, and apoptosis.

### The deficiency of COX-2 promotes *M. bovis* infection by inhibiting ER stress-mediated apoptosis

**Figure 4.**
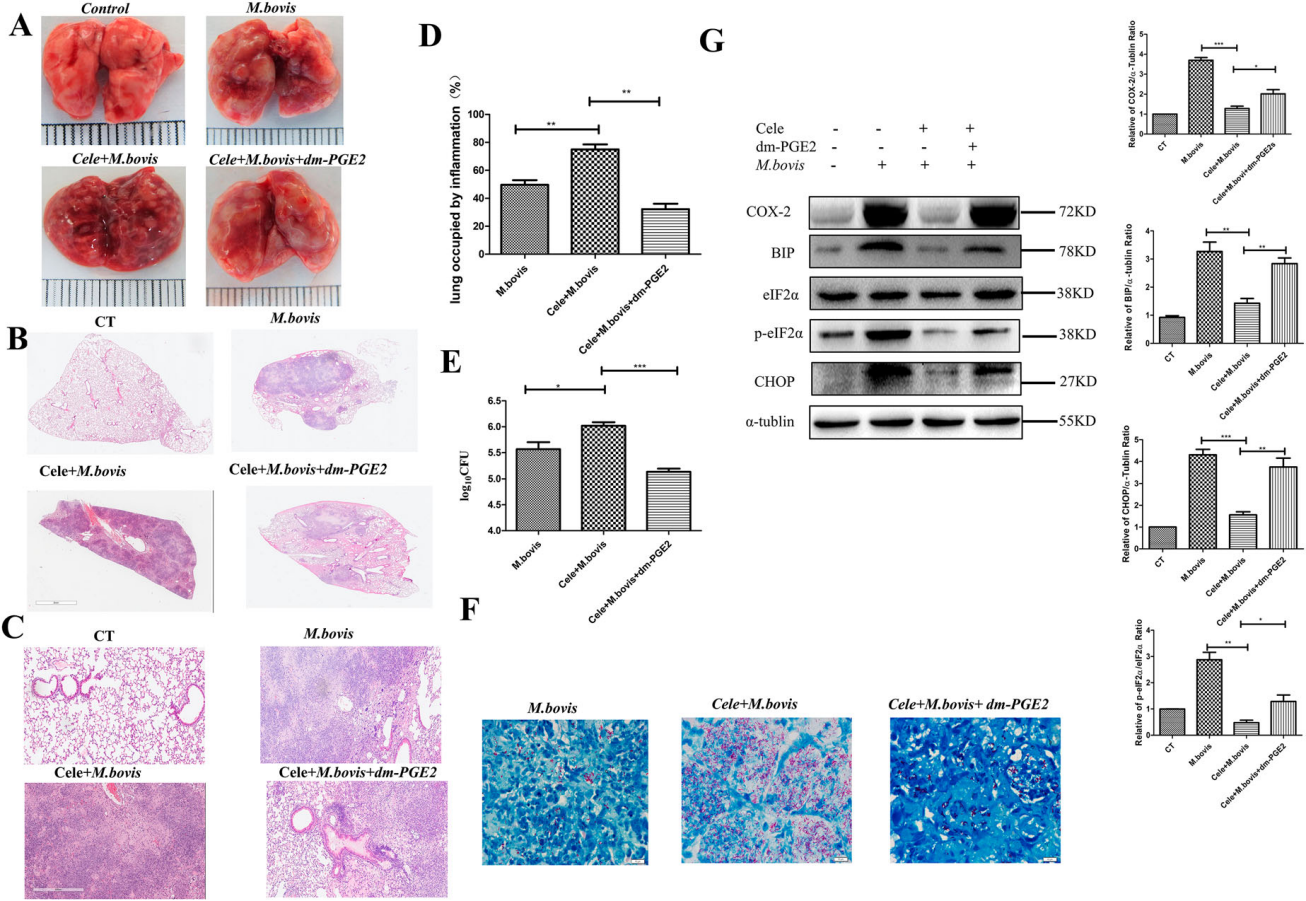
The deficiency of COX-2 promoted *M. bovis* infection by inhibiting ER stress and apoptosis. (A) The representative images of the lungs showed the gross pathological changes in a different group of mice (*n* = 6/group). (B) The representative images of the left lung lobe showed histopathological changes in a different group of mice (*n* = 6). (C) Higher magnification of H&E staining sections of the lungs and spleen showed *M. bovis*-induced lesions. Scale bar: 20 μm. (D) The percentage of the lung’s area occupied by inflammatory lesions was quantified by Image J software. (E) The total number of *M. bovis bacilli* in the lung tissues of infected mice as determined using the CFU assay. (F) Ziehl–Neelsen staining results showing the number of *M. bovis* in the lungs of mice with gut dysbacteriosis. (G) Western blot detection and the relative lung ratio of COX-2, BIP, p-eIF2α, and CHOP in the lungs of *M. bovis*-infected mice, Cele+ *M. bovis* mice and Cele+ *M. bovis + *dm-PGE2. α-tublin and eIF2α expression served as a lung control and was used for normalization (*n* = 3/group). Data are shown as mean ± SD, ***P < 0.001, **P < 0.01. *P < 0.05.

To evaluate the effect of COX-2 in *M. bovis* infection, we infected mice with *M. bovis* with or without a COX-2 inhibitor (Celecoxib). We found that *M. bovis* caused greyish-white nodular nidus could be exaggerated by Celecoxib and alleviated by dm-PGE2 (Figure 4(A)). Consistent with the gross pathological observation, the histopathological observation showed that extensive inflammatory lesions with necrosis and calcification appeared in the lungs of Celecoxib-treated mice with *M. bovis* infection but much fewer inflammatory lesions appeared in the lungs of mice treated with dm-PGE2 (Figure 4(B–D)). In addition, Celecoxib treatment exacerbated bacterial proliferation, leading to an increased bacterial burden and distribution in the lungs, which was reversed by dm-PGE2 injection (Figure 4(E,F)). This result suggested that COX-2 has a beneficial effect on *M. bovis* clearance.

Next, we sought to find the connection between COX-2 and ER stress. We assessed the protein level of COX-2, BIP, p-eIF2α, and CHOP. Compared with mice not treated with Celecoxib, Celecoxib-treated mice had significantly reduced the protein level of COX-2, BIP, p-eIF2α, and CHOP, which was reversed by dm-PGE2 injection (Figure 4(G)). Similarly, we observed that the effect of *M. bovis* infection increases the protein level of COX-2, BIP, and p-eIF2α could be reversed by celecoxib and exacerbated by Zileuton (COX-2 agonist) *in vitro* (Supplement Fig. 2A and Supplement Fig. 3A). It indicated that COX-2 regulated ER stress during infection.

**Figure 5.**
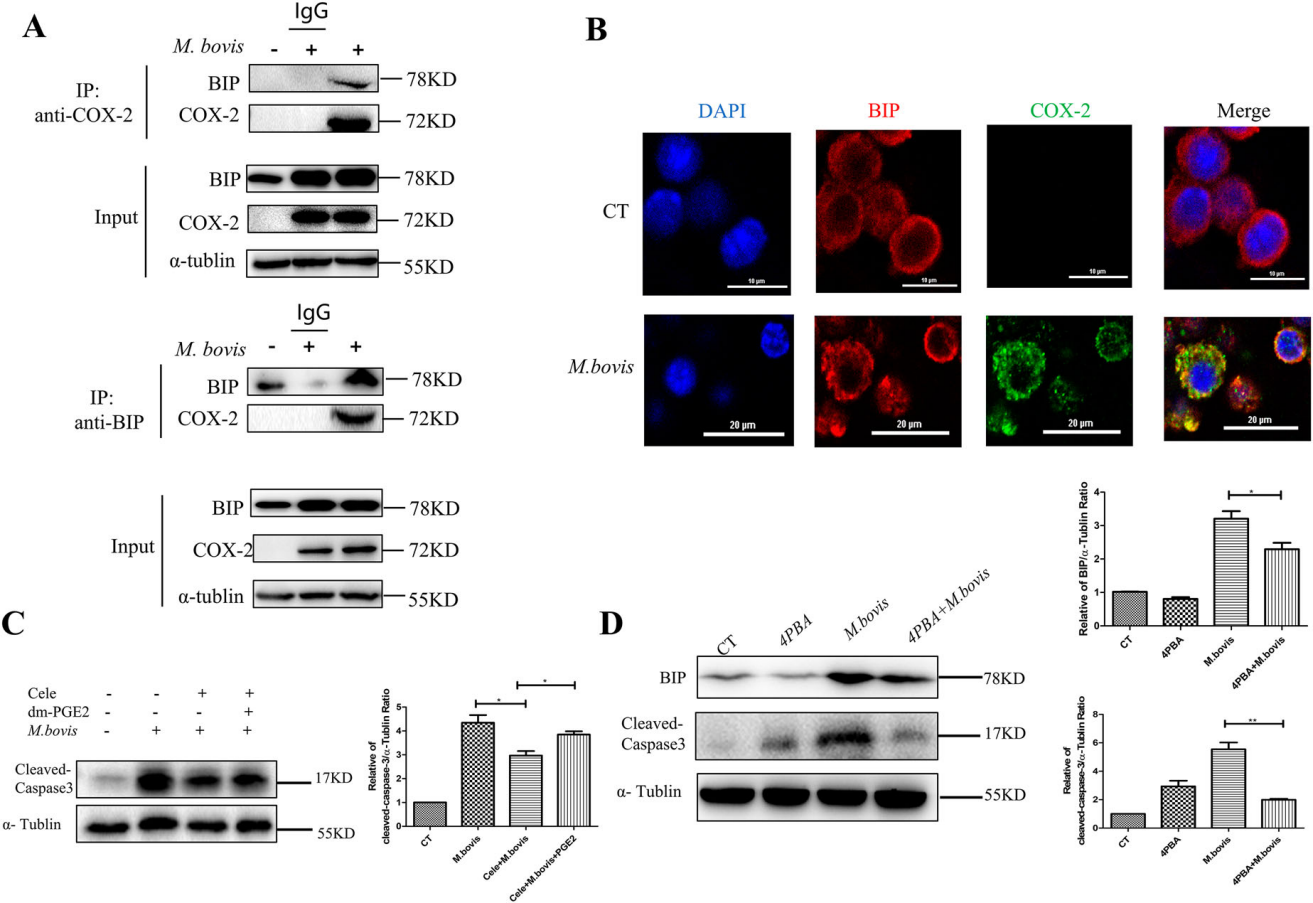
COX-2 interacts with BIP to activate ER stress. (A) Whole-cell lysates were immunoprecipitated with anti-BIP or anti-COX-2 antibody. The total lysates (Input) and IP were immunoblotted with the indicated antibodies. (B) Confocal microscopy analysis of COX-2 (green) colocalization with BIP (red) in RAW264.7 cells infected with *M. bovis* (MOI = 10) for 24 h. (C) Western blot detection and the relative lung ratio of Cleaved -caspase-3 in the lungs of *M. bovis*-infected mice, Cele+ *M. bovis* mice and Cele+ *M. bovis + *dm-PGE2. α-tublin expression served as a lung control and was used for normalization (*n* = 3/group). (D) RAW264.7 were treated with 5 mM 4-PBA for 3 h before infection, then were infected with *M. bovis* (MOI = 10) for 24 h. BIP and Cleaved-caspse-3 were detected by western blot. α-tublin expression served as a cell control and was used for normalization. Data are shown as mean ± SD, **P < 0.01. *P < 0.05.

To confirm the specific mechanism by which COX-2 influences ER stress, we hypothesized that COX-2 is linked to the ER membrane protein BIP. As excepted, the CO-IP experiment revealed an interaction of COX-2 with BIP during *M. bovis* infection (Figure 5(A)). Meanwhile, the confocal microscopy analysis also revealed that with an increase in COX-2 level after *M.bovis* infection, the punctate staining of BIP elevated. The colocalization of COX-2 and BIP rose with *M.bovis* infection (Figure 5(B)). This finding suggests that COX-2 interacts with BIP, while ER stress was activated.

ER stress mediates apoptosis in *Mtb* infection and that is correlated with caspase activation [30,31]. We sought to determine whether COX-2 inhibits apoptosis by the ER stress pathway by assessing the protein level of cleaved-caspase-3. We observed that *M.bovis* infection increased the protein level of cleaved-caspase-3 that was blocked by Celecoxib and enhanced by dm-PGE2 (Figure 5(C)). Meanwhile, we found that fewer apoptotic cells and cleaved-caspase-3 were induced by Celecoxib *in vitro* (Supplement Fig. 2A,2B). Furthermore, compared with cells not treated with Zileuton, Zileuton-treated cells significantly increased the protein level of cleaved-caspase-3 (Supplement Fig. 3A). In addition, enhanced ER folding capacity and alleviated ER stress followed by 4-phenyl butyric acid (4-PBA) pre-treatment could decrease the level of cleaved-caspase-3 and lower *M. bovis*-induced apoptosis (Figure 5(D)), suggesting that COX-2 decreased *M. bovis*-induced apoptosis dependent on ER stress.

### Inhibition of ER stress suppresses COX-2 by NF-kB

**Figure 6.**
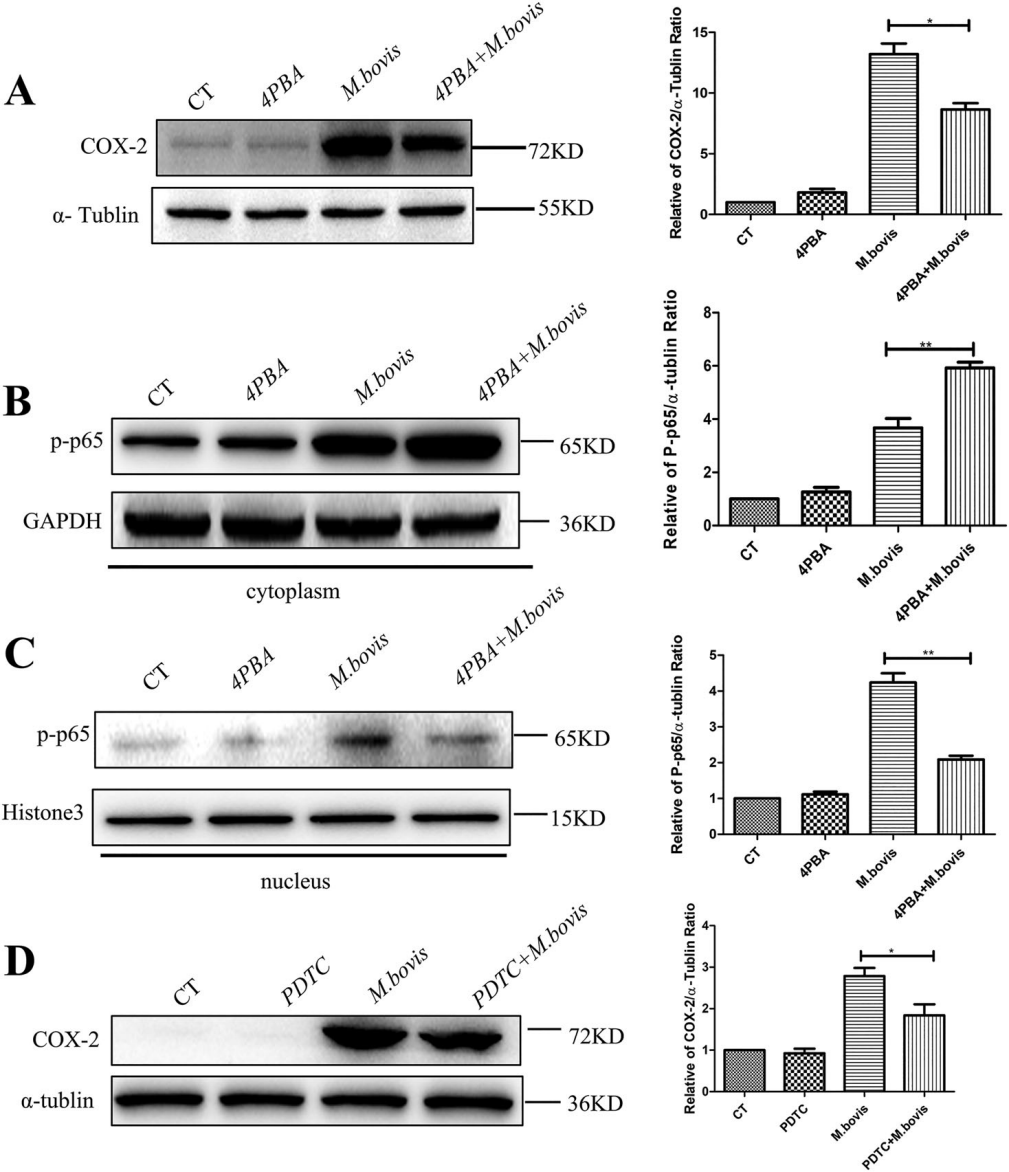
4-PBA inhibited COX-2 through the NF-κB pathway. (A) RAW264.7 were treated with 5 mM 4-PBA for 3 h before infection, then were infected with *M. bovis* (MOI = 10) for 24 h. COX-2 was detected by western blot. α-tublin expression served as a cell control and was used for normalization. (B,C) RAW264.7 was treated with 5 mM 4-PBA for 3 h before infection, then was infected with *M. bovis* (MOI = 10) and treated with or without 4-PBA for 24 h. (B) The expression of p-p65 in the cytoplasm was determined by western blot. GAPDH expression served as a cell control and was used for normalization (C) Expression of p-p65 in the nucleus was determined by western blot. Histone3 expression served as a cell control and was used for normalization. (D) RAW264.7 was treated with 80 μM PDTC for 3 h before infection, then were infected with *M. bovis* (MOI = 10) and treated with or without PDTC for 24 h. Expression of COX-2 in the whole-cell protein was determined by western blot. α-tublin expression served as a cell control and was used for normalization. Data are shown as mean ± SD, **P < 0.01. *P < 0.05.

Intriguingly, we found that the inhibition of ER stress, which was treated by 4-PBA, also reduced the level of COX-2 (Figure 6(A)). This finding implies that a positive feedback regulation exit between ER stress and COX-2. Next, our interest was to explore how ER stress regulates COX-2. We discovered that p-p65, a downstream component of the NF-κB pathway, was significantly increased in the cytoplasm and reduced in the nucleus of the 4-PBA + *M.bovis* treatment group (Figure 6(B,C)), suggesting that ER stress could activate NF-κB pathway. Meanwhile, COX-2 expression was dramatically reduced in *M. bovis*-infected cells treated with PDTC, an NF-κB inhibitor (Figure 6(D)). The results demonstrate that ER stress regulates COX-2 expression by the NF-κB pathway.

## Discussion

Here, we demonstrated the disadvantages of microbiome changes exacerbating *M. bovis* infection and decreasing the level of COX-2. In further research, we found that the lack of COX-2 inhibited ER stress-mediated apoptosis. Next, the Confocal microscopy analysis clearly shows that COX-2 regulated ER stress by interaction with BIP. This finding enriches data on the gut-lung axis and the specific mechanism of COX-2 regulation of ER stress was revealed.

Recent studies are expanding our understanding of gut microbiota, which is the ecosystem of bacteria, archaea, protists, fungi, and viruses that functions not only as an inactive bystander but also as an interconnected and active player with host immunity and diseases [32]. For example, gut dysbacteriosis had significantly reduced mononuclear phagocyte activation and impaired type I IFN production in peripheral blood mononuclear cells (PBMC), spleens, and brains [33]. In addition, chronic antibiotic use caused acute liver failure by destroying the gut barrier and promoting hepatocyte necrosis [34]. Several studies found links between gut microbiota characteristics and tuberculosis in various species. For example, the diversity of intestinal microbiota was decreased in *Mtb*- infected patients. These changes were not significant and only occurred in Bacteroide [35]. In addition, after broad-spectrum antibiotic treatment, *Mtb* growth in the lungs increased, possibly due to a decrease in IFN-γ and TNF-α levels [36]. Another study discovered that gut dysbiosis reduced the efficacy of the *Mtb* vaccine by inhibiting the activation of CD4^+^ T cells and CD8^+^ T cells [37]. Here, we adapted a model of microbiota dysbiosis consisting of the administration of a cocktail of broad-spectrum antibiotics composed of ampicillin, neomycin sulphate, metronidazole, and vancomycin for 7 weeks. This procedure has previously been shown to deplete all detectable commensals and bacterial products [38,39]. Consistent with former studies, our histopathological analysis results revealed that gut dysbacteriosis significantly increased the percentage of inflammation area. Meanwhile, we also found that Abx treatment will cause a heavier bacterium load. Hence, our results indicated that gut dysbacteriosis attenuates resistance to *M. bovis* infection.

Here, we found that COX-2 was almost non-existent before *M. bovis* infection but increased dramatically after infection in macrophages and mice. Also, with *M. bovis* infection, gut dysbacteriosis resulted in lower levels of COX-2 or PGE2 in lungs, guts, and serum, while faecal transplantation reversed this condition. These data provided evidence that the expression of COX-2 or PGE2 may influence *M. bovis* infection. Consist with our results, COX-2 inhibition leads to successful *Mtb* colonization in the lungs during acute infection, whereas COX-2 upregulation leads to improved *Mtb* clearance during chronic infection [40]. However, the role of COX-2 during infection is controversial, but the observation and interpretation of data in different experimental settings appear to be rather heterogeneous. For instance, with conflicting reports, the role of PGE2 on inflammasome and IL-1β activity remains unclear. Previous studies found that inhibiting the NLRP3 inflammasome with PGE2 reduces IL-1β secretion by suppressing the intracellular cAMP-NLRP3 inflammasome pathway [41]. However, other research groups discovered that PGE2 promotes IL-1β secretion by the EP2/EP4 receptor [42]. In summary, the difference in IL-1β expression could be explained by the difference in PGE2 exposure time.

*Mtb* is engulfed by macrophages during infection. Apoptosis occurs when *Mtb* levels in macrophages reach a certain threshold and eliminates *Mtb* [43]. Hence, the survival of bacteria is largely dependent on the innate immunity of the host.

ER stress is a component of innate immunity, which helps to prevent pathogen invasion. For example, excessive production of viral glycoproteins would pose tremendous stress potential on the ER protein-folding mechanism of host cells to affect the host immune during influenza A virus (IAV) infections [44]. In other studies, ER stress was found to increase IFN-γ expression to lower the risk of chronic hepatitis C virus infection [45]. In addition, ER stress activates the STING-TBK1-IRF3 pathway, which induces apoptosis and effectively controls *M. bovis* infection [46]. Here, we discovered that dysregulation of intestinal flora could inhibit the expression of COX-2, BIP, and P-eIF2α. Meanwhile, *M.bovis*-induced increased COX-2, BIP, and P-eIF2α were suppressed by celecoxib and enhanced by zileuton *in vivo* or *in vitro*. Hence, our results indicated that COX-2 regulated the ER stress pathway during *M.bovis* infection.

Recent studies have indicated that the development of acute and chronic kidney disease including renal fibrosis is associated with ER stress. S100 calcium-binding protein 16 (S100A16), a novel member of the S100 family is involved in kidney disease. A significant increase in S100A16 expression in the cytoplasm following renal injury. GRP78 (BIP) then moves into the cytoplasm and binds with S100A16 to activate ER stress [47]. Hence, GRP78 could be transported from the ER to the cytoplasm. In addition, COX-2 had been demonstrated to interact with GRP75, a glucose-regulated protein, to maintain Mitochondria-associated endoplasmic reticulum membranes (MAMs), while GRP75 and BIP are from the same protein family [48]. Based on the results, it is reasonable to suspect the interaction between COX-2 and BIP. Here, the confocal microscopy analysis indicated that the colocalization of COX-2 and BIP was found to rise with *M.bovis* infection, and the CO-IP experiment revealed an interaction of COX-2 with BIP during *M. bovis* infection. Collectively, those results demonstrated that COX-2 interacted with BIP, while ER stress was activated. Additionally, a study found that ER stress regulates COX-2 expression to induce autophagy in cadmium-induced kidney injury [49]. Consistent with our results, ER stress regulated COX-2 levels by the NF-κB pathway. Those results demonstrated the existence of cross-talk between COX-2 and ER stress.

In conclusion, the finding demonstrates that gut dysbacteriosis worsens *M. bovis* infection by lowering systemic COX-2 expression, which inhibits the induction of ER stress-mediated apoptosis. Meanwhile, this study provides new insight into the probable cross-talk of COX-2 and ER stress. We believe that using celecoxib during TB treatment may exacerbate the disease process. Collectively, these findings provide a theoretical foundation for controlling the *mycobacterium* infection process and developing therapeutic strategies.

## Supplementary Material

Supplemental Material

## Data Availability

All data generated or analyzed during this study are included in this published article.
