# Supplementary material for: Gut dysbacteriosis attenuates resistance to Mycobacterium bovis infection by decreasing cyclooxygenase 2 to inhibit endoplasmic reticulum stress
Source: Emerg Microbes Infect. 2022 Jul 21;11(1):1806–18. doi: 10.1080/22221751.2022.2096486 (PMC9307115; doi:10.1080/22221751.2022.2096486)
Supplement: Supplemental Material [file TEMI_A_2096486_SM8189.zip › supplement table.docx]

**Primers used in this study.**

| **Primer name and sequence** | **(5′-3′ )** |
| --- | --- |
| - Bifidobacterium(forward) | CGGGTGAGTAATGCGTGACC |
| - Bifidobacterium（reverse) | TGATAGGACGCGACCCCA |
| - Bacteroides(forward) - Bacteroides（reverse) | GAGAGGAAGGTCCCCCAC  CGCTACTTGGCTGGTTCAG |
| - Lactobacillus(forward) - Lactobacillus（reverse) - Enterococcus(forward) - Enterococcus（reverse) - Campylobacter(forward) - Campylobacter（reverse) - Total bacteria(forward) - Total bacteria（reverse) | AGCAGTAGGGAATCTTCCA  CACCGCTACACATGGAG  CCCTTATTGTTAGTTGCCATCATT  ACTCGTTGTACTTCCCATTGT  GGATGACACTTTTCGGAG  AATTCCATCTGCCTCTCC  ACTCCTACGGGAGGCAGCAGT  ATTACCGCGGCTGCTGGC |
