# Supplementary material for: Gut dysbacteriosis attenuates resistance to Mycobacterium bovis infection by decreasing cyclooxygenase 2 to inhibit endoplasmic reticulum stress
Source: Emerg Microbes Infect. 2022 Jul 21;11(1):1806–18. doi: 10.1080/22221751.2022.2096486 (PMC9307115; doi:10.1080/22221751.2022.2096486)
Supplement: Supplemental Material [file TEMI_A_2096486_SM8189.zip › supplement_figure_legends.docx]

**Supplement figure1**. Construction of animal experimental model. (A) C57BL/6 Mice were pre-treated with broad spectrum of antibiotics for 3 weeks. Then C57BL/6 mice were infected with *M. bovis* at 200 CFU via the intranasal route and continued treatment with antibiotics for 5weeks. (B) C57BL/6 mice were infected with *M. bovis* at 200 CFU via the intranasal route. Meanwhile, mice were treated with DMSO (n = 5) (10%) or Celecoxib (20μg/kg) (n = 5) or Dimethyl PGE2 (50 μg/ml per mouse) (n = 5) via intraperitoneal (i.p.) injection for 5weeks. On days 5weeks after infection, animals were sacrificed and different organs were collected. (C) the body weight of mice was calculated in all experimental groups on weekly; ‘+’ means infection *M.bovis.*(D) Representative images of spleen’s tissues for gross lesion studies from various groups (n=6).

**Supplement figure2**. Celecoxib inhibited apoptosis by suppressing ER-Stress in vitro

(A) RAW264.7 was treated with Annexin-V and detected by flow cytometry. Statistical analysis of apoptotic cells, n=3, *p<0.05, **p<0.01. (B）RAW264.7 were treated with 30μM Celecoxib for 3 h prior to infection. Then it infected with *M. bovis* (MOI = 10) Celecoxib for 24 h. Western blot detection of COX-2, p-eIF2α, BIP, Cleaved-caspase3 and in lung of *M. bovis*-infected mice and Cele+ *M. bovis* mice. α-tublin and eIF2α expression served as aRAW263.7 control and was used for normalization. (n = 6/group). Data are shown as mean ± SD **P < 0.01. *P <0 .05.

**Supplement figure3**. Zileuton activated apoptosis by regulating ER-Stress in vitro (A) RAW264.7 were treated with 20μM Zileuton for 3 h prior to infection. Then it infected with *M. bovis* (MOI = 10) and Zileuton for 24 h. Western blot detection of COX-2, p-eIF2α, BIP, Cleaved-caspase3 and relative intensity ratios with α-Tublin and eIF2α normalization in RAW264.7 (n = 6/group). Data are shown as mean ± SD, **P < 0.01. *P < 0.05.
