# Supplementary figures and images for: Gut dysbacteriosis attenuates resistance to Mycobacterium bovis infection by decreasing cyclooxygenase 2 to inhibit endoplasmic reticulum stress
Source: Emerg Microbes Infect. 2022 Jul 21;11(1):1806–18. doi: 10.1080/22221751.2022.2096486 (PMC9307115; doi:10.1080/22221751.2022.2096486)

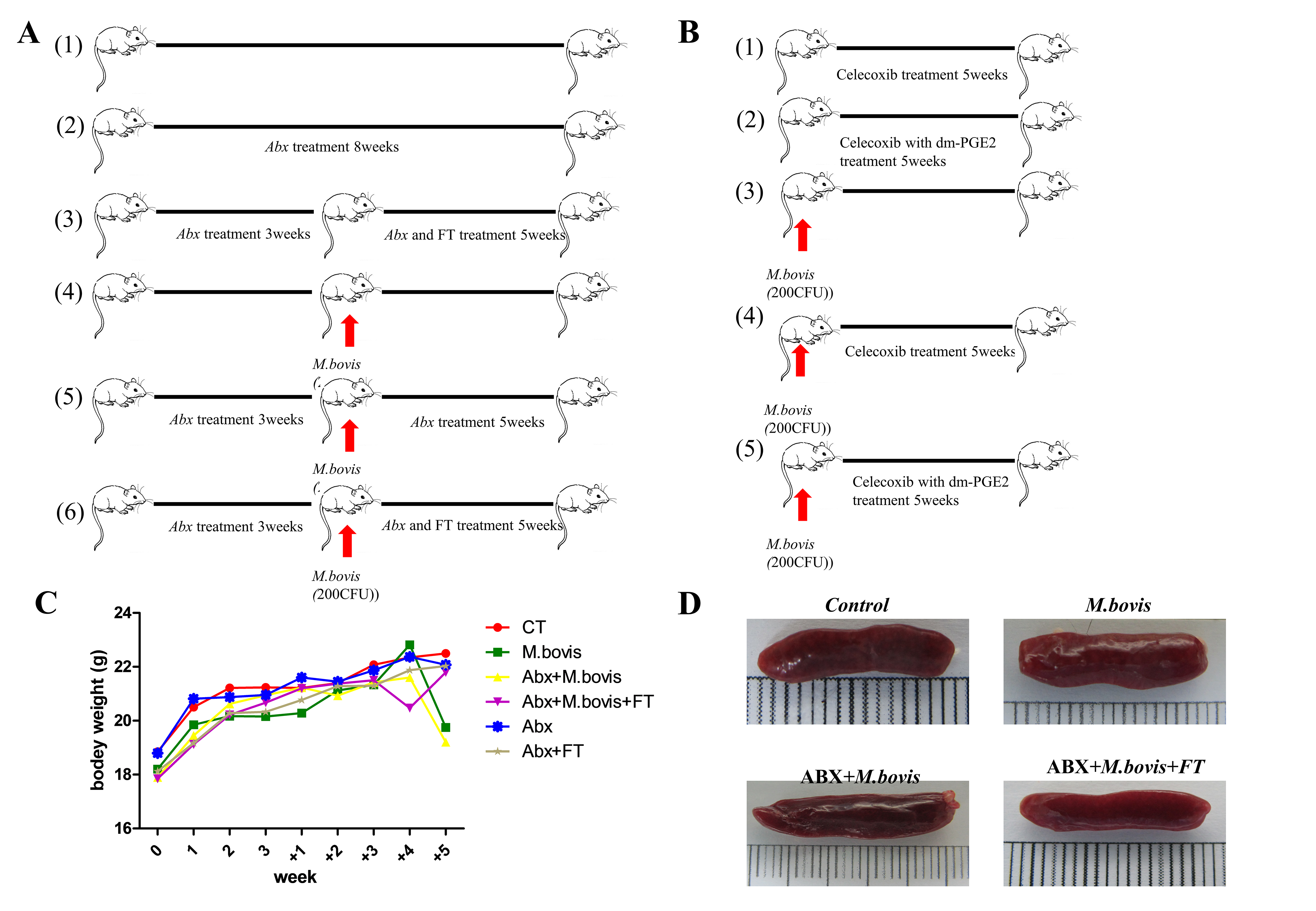

Supplement: Supplemental Material [file TEMI_A_2096486_SM8189.zip › sup figure1.tif]

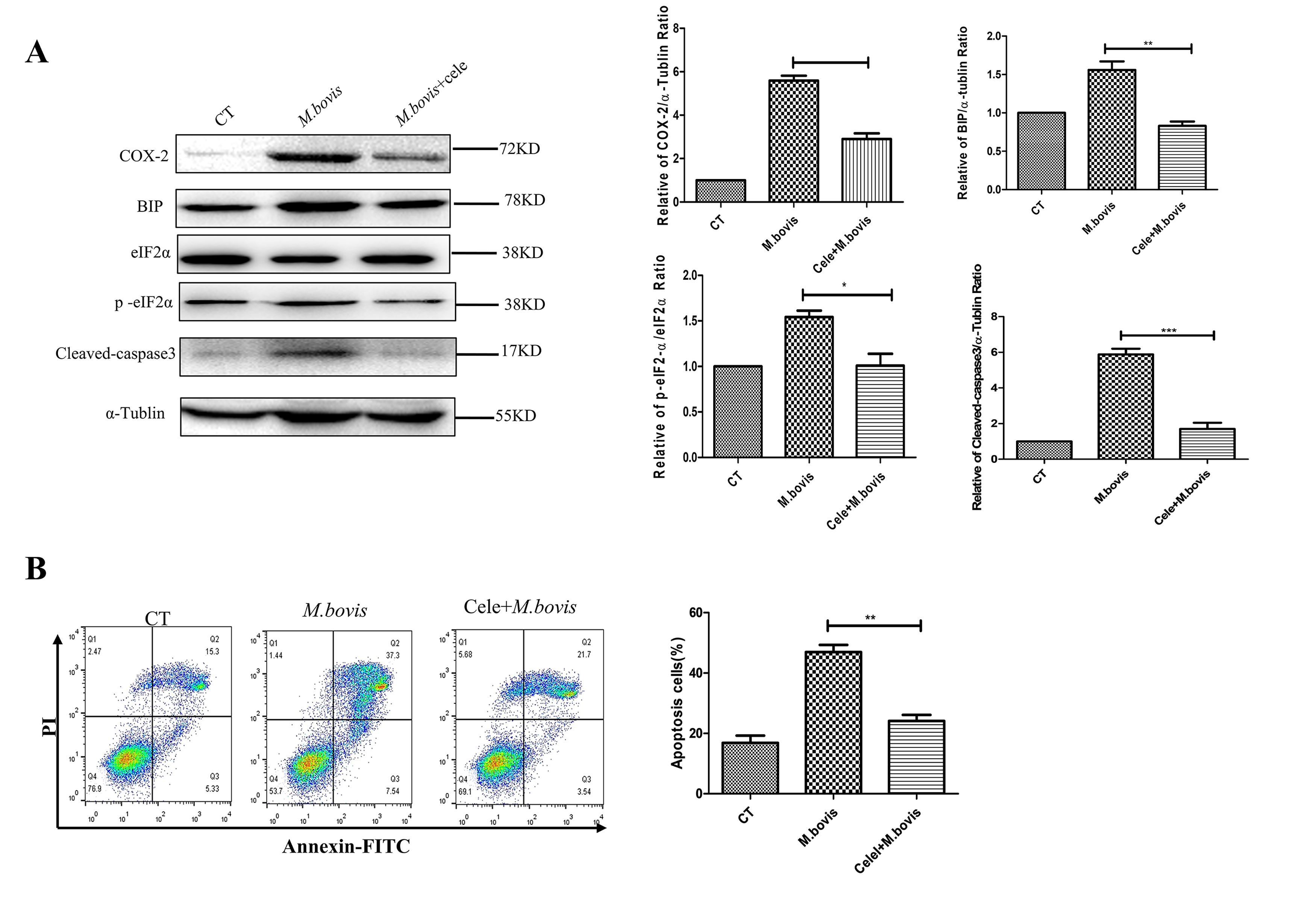

Supplement: Supplemental Material [file TEMI_A_2096486_SM8189.zip › sup figure2.tif]

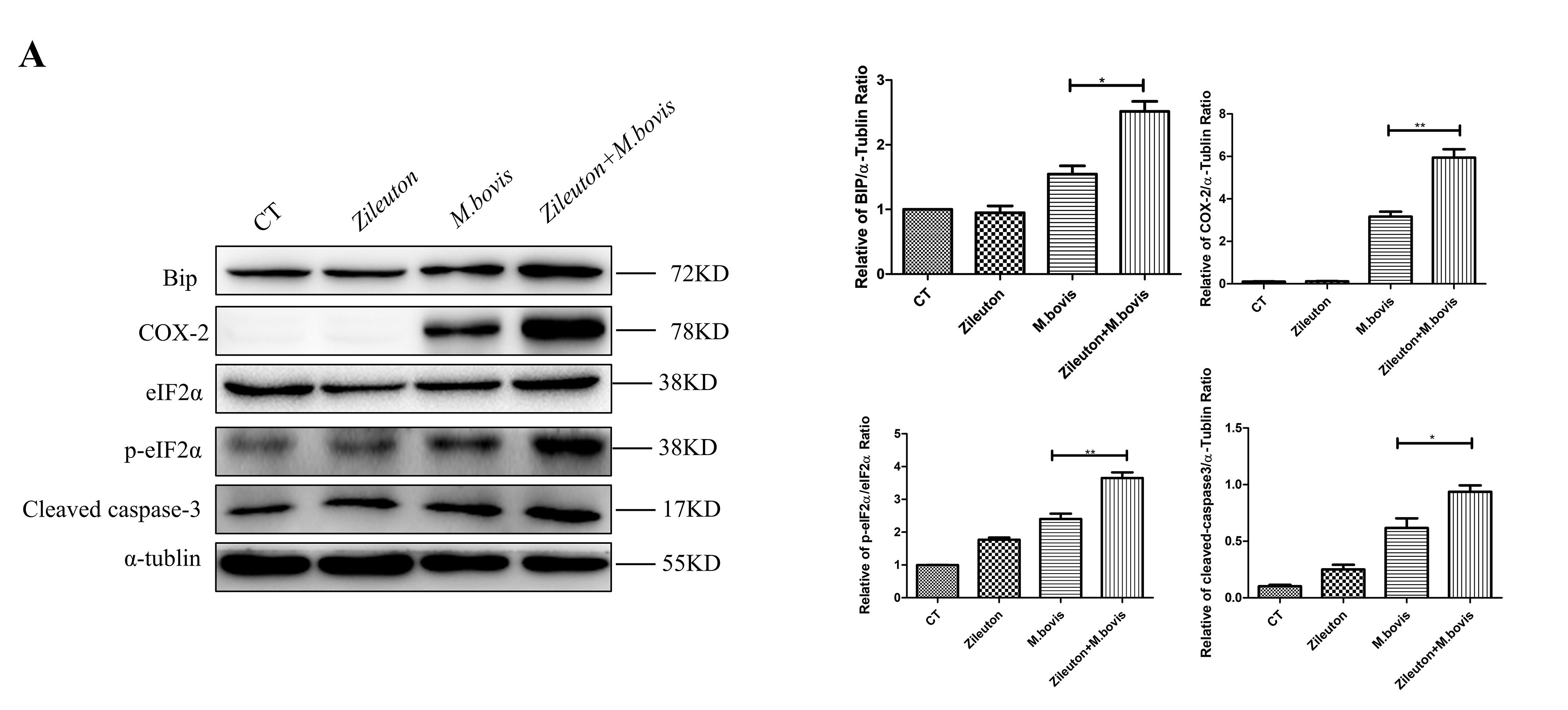

Supplement: Supplemental Material [file TEMI_A_2096486_SM8189.zip › sup figure3.tif]
